# Reliability and repeatability of a modified thoracolumbar spine injury classification scoring system

**DOI:** 10.3389/fsurg.2022.1054031

**Published:** 2023-01-06

**Authors:** Wen-jie Lu, Jiaming Zhang, Yuan-guo Deng, Wei-yu Jiang

**Affiliations:** ^1^ Department of Spine Surgery, Ningbo No. 6 Hospital, Ningbo, China; ^2^ The Second Clinical Medical School of Zhejiang Chinese Medicine University, Hangzhou, China

**Keywords:** thoracolumbar fracture, severity, scoring, reliability and repeatability, modified typing

## Abstract

**Purpose:**

On the basis of the Thoracolumbar Injury Classification and Severity Score (TLICS), an modified TLICS classification system was presented, its reliability and repeatability were assessed, and the factors influencing classification consistency were examined.

**Methods:**

Five spinal surgeons were chosen at random. The clinical data of 120 patients with thoracolumbar fractures admitted to the Department of Spine Surgery, Ningbo Sixth Hospital from December 2019 to June 2021 were categorized using the modified TLICS system. After 6 weeks, disrupt the order of data again. Using unweighted Cohen's kappa coefficients, the consistency of the modified TLICS system was assessed in five aspects: neurofunctional status, disc injury status, fracture morphology, posterior ligament complex (PLC) integrity, and treatment plan.

**Results:**

In terms of reliability, the average kappa values for the subclasses of the modified TLICS system (neurofunctional status and disc injury status) were 0.920 and 0.815, respectively, reaching the category of complete confidence. Fracture morphology and treatment plan had average kappa values of 0.670 and 0.660, respectively, which were basically reliable. The average kappa value of PLC integrity was 0.453, which belonged to the category of moderate confidence. The average kappa coefficients of each subcategory (neurological status, disc injury status) had excellent consistency, and the kappa values were 0.936 and 0.879, respectively, which belonged to the completely credible category. The kappa values of fracture morphology and treatment plan repeatability were 0.772 and 0.749, respectively, reaching the basic credibility category. PLC integrity repeatability kappa value is low, 0.561, to moderate credibility category.

**Conclusion:**

The modified TLICS system is intuitive and straightforward to understand. The examination of thoracolumbar fracture injuries is more exhaustive and precise, with excellent reliability and repeatability. The examination of neurological status and disc injury status is quite reliable and consistent. The consistency of fracture morphology is slightly poor, which is basically credible; the PLC integrity consistency is poor, reaching a reliability level of moderate, which may be associated with the subjectivity of clinical evaluation of PLC.

## Background

Thoracolumbar fractures often refer to injuries of the T11-L2 segment, which is the most common type of spinal injury and is frequently caused by direct trauma, accounting for about 80% of spinal injuries (1). Due to the unique anatomical location and characteristics of the thoracolumbar segment, the clinical manifestations and treatment after injury differ from those of thoracic and lumbar fractures. If timely diagnosis and treatment are not obtained, or if the treatment method chosen is unreasonable, it is not conducive to improving the long-term quality of life of patients (2, 3). The classification of thoracolumbar fractures is important for clinical treatment and prognosis. At present, the Denis, AO, and TLICS system are the most prevalent staging approaches. However, the Denis classification is too basic to cover all fracture types and has limited clinical significance (4). The AO classification is quite complex, and its therapeutic application is difficult to learn. In the meantime, the average kappa value for confidence is 0.517, and the kappa values for each subtype are lower, placing them in the category of low to moderate confidence (5, 6). For the first time, the TLICS classification considers fracture morphology, PLC integrity, and neurofunctional status as key variables in assessing fracture damage severity and guiding physicians in selecting whether to pursue surgical intervention and how to select the surgical method. In recent years, numerous academics have evaluated the reliability and repeatability of the TLICS classification system, and research indicates that it may be the most reliable and effective classification system for the current clinical evaluation of the treatment of thoracolumbar fractures (7–9). Later, as MRI technology and spinal biomechanics progressed, researchers became increasingly worried about the influence of intervertebral disc and ligament structural integrity on the stability of the spine. Changes in spinal structure following a fracture can be categorized as bone structure changes or non-bone structure changes. Existing categorization approaches focus mostly on bone structure changes. Although the TLICS method considers PLC integrity, the impact of intervertebral disc damage on spinal stability was not examined. In addition, due to limits in technical progress and a multitude of influencing factors, PLC integrity cannot be assessed reliably, compromising the consistency of the TLICS system (10–12). Consequently, we presented a modified TLICS system, which included the evaluation of “disc injury status” and a reduction in the score for “PLC integrity.” This study aims to recruit 120 patients with thoracolumbar fractures admitted to the Spinal Surgery Department of the Sixth Hospital of Ningbo City between December 2019 and June 2021 to examine the reliability and repeatability of the modified TLICS system, investigate the clinical guiding significance of the system, and investigate the factors affecting the system's consistency.

## Materials and methods

### General information

Patients have been diagnosed with fresh single-stage traumatic thoracolumbar fractures and no other serious injuries or illnesses. In addition, complete clinical imaging data and informed consent signed by patients and their families were also necessary. Thoracolumbar fractures that were multi-level or have been there for more than 3 weeks should be ruled out. Patients with osteoporotic fractures, severe multiple trauma, such as a head injury, and missing or incomplete imaging data should be excluded as well.

The study comprised a total of 120 patients with thoracolumbar fractures, including 68 males and 52 females. Imaging data included preoperative anteroposterior and lateral thoracolumbar x-ray, CT, and MRI. All data did not contain any information and markers related to the classification. The study was authorized by the Ethics Committee of Ningbo Sixth Hospital, and all procedures were conducted in accordance with applicable rules and standards. All patient-related information was authorized for publication by the patients or their legal guardians.

### Research method

**Figure 1:**
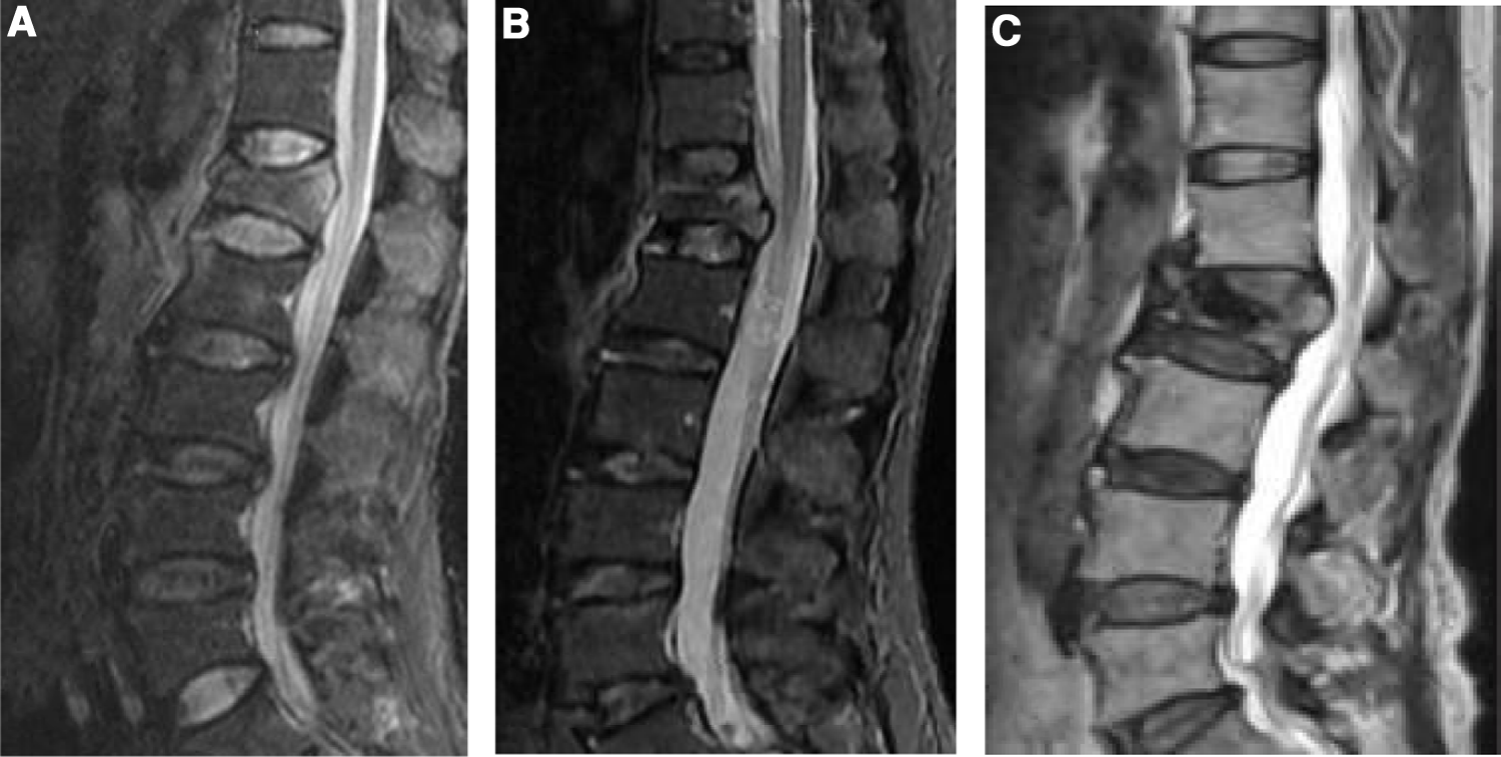
According to the imaging characteristics of disc injury, it was divided into three categories: no injury, mild injury, and moderate-to-severe injury (sagittal MRI images of the thoracolumbar segment before treatment). (**A**) No intervertebral disc injury (0 points). (**B**) Mild intervertebral disc injury, signal change, no endplate injury, with or without space change (1 point). (**C**) Moderate-to-severe intervertebral disc injury, significant signal change, end plate fracture, intervertebral disc contents herniated into vertebral body, intervertebral space change (2 points).

**Figure 2:**
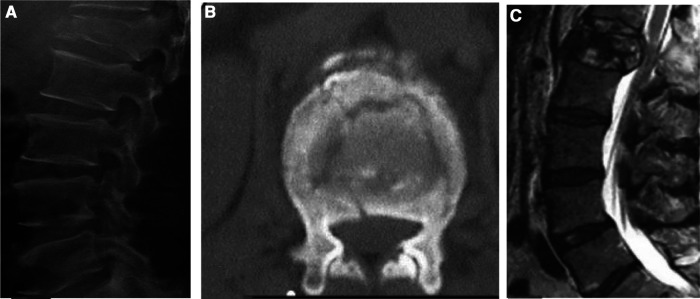
Patient, a male, 52 years old, was hospitalized for 1 day with low back pain due to trauma, no neurological symptoms, and was diagnosed with an L1 burst fracture. (**A,B**) burst fracture of L1 vertebral body, no neurological injury; (**C**) L1 burst fracture, no neurological injury, suspicious injury of PLC status, severe disc injury status. **The modified TLICS system:** burst fracture (2 points), suspicious PLC injury (1 point), severe disc injury status (2 points), no neurological injury (0 points), T = 5 points, surgical treatment is recommended. **The TLICS system:** T = 4 points; treatment choices are recommended according to the patient's specific situation, and there are differences between them.

**Figure 3:**
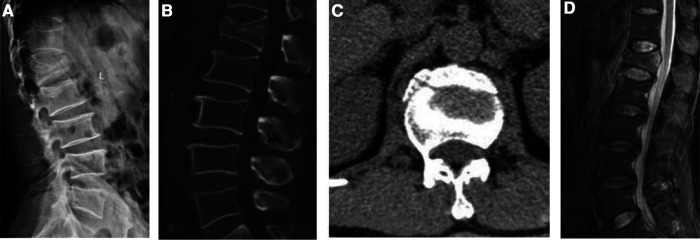
Patient, a male, 44 years old, was hospitalized for 2d for traumatic back pain, no neurological symptoms, diagnosed as L1 burst fracture. (**A–C**) suggesting an L1 burst fracture with no nerve damage; (**D**) an L1 burst fracture with no damage to the PLC, no damage to the intervertebral disc, and no neurological injury. **The modified TLICS system:** burst fracture (2 points), no damage to the PLC (0 points), no damage to the intervertebral disc (0 points), no neurological injury (0 points), T = 2 points, Non-surgical treatment is recommended based on the modified TLICS system.

This study proposed a modified TLICS system to add the subcategory of “disc injury status” and lower the score of “PLC integrity” based on the clinical data collected, previous domestic and foreign literature publications, and the TLICS system. The subcategory of “disc injury status” should be analyzed in conjunction with MRI imaging data, classified into no injury, mild injury, and moderate-to-severe injury based on imaging characteristics of disc injury, and assigned 0, 1, and 2 points, respectively (Figure 1). The score assigned to the “PLC integrity” subcategory was appropriately reduced, with 0 points assigned when there was no injury to the PLC, 1 point assigned when there was a suspicious injury to the PLC, and 2 points assigned when there was an injury. The overall score of fracture morphology, PLC integrity, neurofunctional state, and disc damage status then guided clinical therapy and prognosis (Table 1). When the total score was T < 4, non-surgical treatment was administered; when the total score was T = 4, either non-operative or surgical treatment was administered; and when the total score was T > 4, surgery was performed (Figures 2, 3). Two associate chief physicians and three attending physicians were chosen at random and instructed using the modified TLICS system. After completing the training, five physicians categorized and rated the imaging data of five patients with thoracolumbar fractures to assess the mastery of the scoring system. After the test was qualified, the medical image data of 120 patients were independently scored by 5 physicians and allowed to refer to the original literature. After 6 weeks, disrupted the order of data again classification score. The results of the two classifications were recorded by physicians who were not involved in the classification, and the correlation between the final total score T and the choice of treatment plan was analyzed. Then the reliability and repeatability of the modified TLICS system were analyzed to explore the reasons for the consistency of the classification. For the same patient, 5 physicians in classification were inconsistent as long as there was a physician classification of any sub-category score that was different.

**Table 1:** Modified TLICS staging scoring system.

Subcategory/system score	TLICS System	Modified TLICS system
Fracture morphology		
Compressive	1	1
Bursting	2	2
Reduced force and rotational	3	3
Distraction	4	4
Neurofunctional status		
No injury	0	0
Nerve root injury	2	2
Complete spinal cord/Conus injury	2	2
Incomplete spinal cord/Conus injury	3	3
Cauda equina injury	4	4
PLC integrity		
No injury	0	0
Uncertain	2	1
Disruption	3	2
Disc injury status		
No damage		0
Mild injury		1
Moderate-to-severe injury		2

### Observation items and methods

At first, a correlation study was performed to clarify the relationship between the scores of the modified TLICS system and the patient's final treatment plans. The consistency of the modified TLICS system was then assessed. The observation indicators included the scores of each subtype supplied by the modified TLICS system at two stages before and after five physicians, and the scores of each subtype among five physicians were statistically added at two stages before and after five physicians. According to the reliability evaluation criteria of Landis and Koch, the reliability was analyzed by the consistency kappa test, and the repeatability of two-stage categorization scores before and after the same doctor was studied.

### Statistical processing

The correlation between modified TLICS system scores and patient treatment regimens was analyzed by Pearson correlation analysis with a test level of *α* = 0.05, and *P* < 0.05 indicates that the difference is statistically significant. An inter-observer consistency test (reliability analysis) was conducted for intra-group typing data, an intra-observer consistency test (repeatability analysis) was performed on the inter-group typing data, and SPSS 26.0 software was used to calculate the kappa coefficient. The degree of consistency was judged according to the Landis and Koch (11) classification system. When Kappa value >0, it is meaningful. The greater the Kappa coefficient, the better the reliability or repeatability. When the Kappa value is 0.00–0.20, it indicates poor consistency and belongs to the category of mild credibility; when the Kappa value is 0.21–0.40, it indicates general consistency and belongs to the category of mild to moderate credibility; When the Kappa value is 0.41–0.60, it indicates moderate consistency and belongs to moderate credibility category; When the Kappa value is 0.61–0.80, it shows good consistency and belongs to the basic credible category; when the Kappa value is 0.81–1.00, it shows excellent consistency and is completely credible.

## Results

A total of 120 patients with thoracolumbar fractures were included in this study, comprising 68 men and 52 females, and aged 22∼65 (36.7 ± 5.7) years old. The demographic information is displayed in Table 2. Among the 120 patients, based on the scores obtained from the modified TLICS system, combined with the systemic conditions and personal wishes, 38 patients had a total score of T < 4, of which 30 were treated conservatively and 8 surgically; 17 patients had a total score of T = 4, of which 5 were treated conservatively and 12 surgically, and 65 patients had a total score of T > 4, of which 4 were treated conservatively and 61 surgically. A correlation analysis of the patients' modified TLICS system score T with the treatment plan revealed a Pearson correlation coefficient of 0.688 and a strong correlation between the two (Table 3).

**Table 2:** General information of patients.

General Information	Number of people (cases)
Sex	
Male	68
Female	52
Age	36.7 ± 5.7
Fracture segment	
T11	14
T12	45
L1	54
L2	27
ASIA Grading	
A	3
B	8
C	12
D	28
E	69
Cause of injury	
Traffic Accidents	63
Crushing by weight	27
Falling from height	18
Other reasons	12

**Table 3:** Correlation of modified TLICS system scores with the treatment plan.

		Total Score	Treatment plan
Total Score	Pearson Correlation	1	0.688
	Sig. (bobtail)		0.000
	Number of cases	120	120
Treatment plan	Pearson Correlation	0.688	1
	Sig. (bobtail)	0.000	
	Number of cases	120	120

Five physicians graded 1,200 times 120 individuals with thoracolumbar fractures(120 cases*5 individuals*2 times). The modified TLICS system's reliability kappa coefficients for each subclass (neurofunctional status and disc injury status) demonstrated excellent consistency across the two classifications, with neurofunctional status having kappa values of 0.903 and 0.936 and disc injury status having kappa values of 0.842 and 0.788, both of which fell into the category of being completely credible. The fracture morphology reliability ratings were 0.660 and 0.698, and the treatment plan reliability scores were 0.625 and 0.694; both scores fell into the basic reliability category. The kappa value of PLC integrity reliability was somewhat low (0.417 and 0.488, respectively), placing it in the moderate reliability category. The repeatability kappa value of each subcategory (neurofunctional status and disc injury status) demonstrated excellent consistency, with kappa values of 0.936 and 0.879, respectively, belonging to the category of being completely credible. The repeatability kappa values for fracture morphology and treatment plan were 0.77 and 0.74, respectively, placing them in the basic credible category. The repeatability kappa value for PLC integrity was low, 0.561, placing it in the moderate credibility category (Tables 4, 5).

**Table 4:** Comparison of reliability and repeatability of kappa values for each subtype and treatment of modified TLICS system by five physicians.

Physicians	Fracture morphology		Neurofunctional status		PLC integrity		Disc injury status		Treatment plan	
	Rel	Rep	Rel	Rep	Rel	Rep	Rel	Rep	Rel	Rep
1	0.663	0.782	0.915	0.927	0.418	0.548	0.819	0.891	0.656	0.745
2	0.608	0.728	0.927	0.921	0.397	0.478	0.767	0.831	0.564	0.691
3	0.716	0.788	0.901	0.948	0.477	0.586	0.782	0.855	0.644	0.777
4	0.697	0.765	0.930	0.948	0.513	0.612	0.864	0.927	0.727	0.805
5	0.711	0.797	0.927	0.936	0.460	0.581	0.843	0.891	0.709	0.727

Rel, Reliability; Rep, Repeatability.

**Table 5:** Reliability and repeatability of each subtype of the modified TLICS system.

Subcategory	The first time	The second time	Kappa value
Fracture morphology	Rel	0.660	0.698	0.679
	Rep	—	—	0.772
Neurofunctional status	Rel	0.903	0.936	0.920
	Rep	—	—	0.936
PLC integrity	Rel	0.417	0.488	0.453
	Rep	—	—	0.561
Disc injury status	Rel	0.842	0.788	0.815
	Rep	—	—	0.879
Treatment plan	Rel	0.625	0.694	0.660
	Rep	—	—	0.749

Rel, Reliability; Rep, Repeatability.

## Discussion

### Necessity and theoretical basis for the proposed modified TLICS system

The thoracolumbar segment of the spine (T11-L2) is a structural transition zone from the thoracic to the lumbar spine, with the articular facets gradually shifting from the coronal to the sagittal plane and a dramatic increase in spinal mobility; its unique anatomical structure and stress mechanism are intrinsic factors in the high incidence of spinal injuries in the thoracolumbar segment. Thoracolumbar fractures are frequently accompanied with spinal nerve damage, which has a high rate of disability and negatively impacts patients' daily lives and quality of life. Consequently, its therapeutic care is very crucial (12-14). The treatment plan for thoracolumbar fractures is primarily determined by assessing spinal stability, and non-operative treatment is typically selected for stable thoracolumbar segment fractures; surgical treatment is selected for unstable fractures to prevent the deterioration of neurological function and the development of secondary symptomatic spinal deformities (15–17). But, the academic community lacks a unified standard for measuring spinal stability, and the thoracolumbar fracture classification system currently in use has significant problems. For example, the Denis classification system is overly simplistic, and its method for distinguishing between stable and unstable fractures is suspect and unreliable. The AO classification system consists of 53 subcategories that are complicated, difficult to remember, and limited in their repeatability. According to research on reliability, the reliability of AO between main kinds is about 67%, and it is considerably lower within subtypes, which have limited clinical practice guiding significance. In addition, the classification lacks a defined definition of “stability” and excludes neurofunctional status as a criterion of scoring (18). The TLICS classification system is a frequently utilized thoracolumbar fracture scoring system; nevertheless, various spinal surgeons may have varying opinions regarding the integrity of the PLC, and the influence of intervertebral disc damage on spinal stability is not taken into account. Therefore, there is a need for a simple and practicable scoring system that considers the immediate stability, long-term stability, and nerve stability of the spine in order to effectively identify the degree of fracture injury, direct therapeutic therapy, and predict prognosis.

### Characteristics of the modified TLICS system

The features of our proposed modified TLICS type system are as follows: (1) In recognition of the scientific character of the TLICS type system, the subcategories “fracture morphology” and “neurofunctional status” were retained to signify, respectively, the immediate stability and neurological stability following spinal fractures. (2) Although the TLICS system takes into account the effect of PLC integrity on the long-term stability of the spine, it is sometimes difficult to assess the PLC's integrity properly in clinical practice. It frequently necessitates the subjective evaluation of patient symptoms and clinical experience by physicians. In this study, the reliability and repeatability kappa values of the “PLC integrity” subcategory were 0.453 and 0.561, respectively, which only reached the moderate confidence category. At the same time, the PLC only bears a large tension load when the spine is subjected to flexion deformity stress, and the anterior and middle spinal structures are more important in maintaining the axial forces in the spine, which bear 70%–80% of the axial compressive stress in the spine and are the most important anatomical structures for maintaining spinal stability. Spinal kyphosis and mechanical instability are mostly due to a lack of support in the anterior and middle columns rather than insufficient posterior column support strength. Therefore, assessing PLC integrity separately from vertebral bony structural damage may lead to an overemphasis on the role of the posterior column (19–21). Therefore, the TLICS system was changed so that “PLC integrity” got a lower score to make it more reasonable. Therefore, the modified TLICS system reduced the score assigned to “PLC integrity” to make it more reasonable. (3) The modified TLICS system classified “disc injury status” as no injury, mild injury, and moderate-to-severe injury. This was the first time that disc injury status was included in the system. This was reasonable because it focused more on how stable the spine will be in the long run.

### Analysis of the reliability and repeatability of the modified TLICS system

The result of this study demonstrated that the modified TLICS system's subcategories for neurofunctional status and disc injury status had excellent consistency. The average kappa values of the subcategories of fracture morphology and treatment plan could reach the basic credible category, while the average kappa value of PLC integrity could only reach the category of moderate confidence. Combining the results of a previous multicenter TLICS system consistency study (22) (fracture morphology, neurofunctional status, PLC integrity, and treatment plan, with average kappa values of 0.430, 0.850, 0.470, and 0.290 for reliability and 0.590, 0.900, 0.550, and 0.440 for repeatability, respectively), we found that both systems had comparable consistency in the neurofunctional status and PLC integrity subcategories, reaching the full and moderate levels of agreement, respectively. When it came to fracture morphology and treatment plan subcategories, the modified TLICS system had better consistency than the TLICS system. Therefore, we had grounds to infer that the modified TLICS system had better consistency than the TLICS system and was more favorable to the clinical diagnosis and treatment of thoracolumbar fractures. Due to the absence of a direct comparison between the two categorization systems in this study, there were several unpredictable variables, including physicians' varying mastery of the classification system and patients' varying acceptance of surgical therapy. To compare the consistency of the two kinds, further controlled research is required.

### Exploration of factors affecting the consistency of the modified TLICS system

#### Reducing the influence of the “PLC status” subcategory assignment

The modified TLICS system assessed each subcategory of fracture morphology, neurofunctional status, disc injury status, and PLC integrity independently, and the cumulative total score determined the treatment plan. From the two classifications, it was evident that the consistency of PLC integrity subcategories was low, and the kappa values of reliability and repeatability were 0.453 and 0.561, respectively, which had a significant impact on the final choice of treatment plan, which may be associated with the accurate assessment of PLC injury status and unreasonable assignment. The PLC consists of the supraspinous ligament, the interspinous ligament, the ligamentum flavum, and the facet joint capsule. It is responsible for the everyday biomechanical actions of the spine, together with the anterior and middle columns, in order to preserve spine stability. Physical examination, x-rays, computed tomography(CT), magnetic resonance imaging (MRI), etc, are currently the most used clinical procedures for determining the extent of PLC damage. When a patient is obese, for instance, the diagnosis may be missed due to an inability to reach the spinous process; when there is bleeding in the spinal canal, it is easy to produce the appearance of a ligamentum flavum injury. Many studies reported similar issues, Zhang Yang et al. (23) discovered that the sensitivity, specificity, and accuracy of physical signs to examine PLC injury were low, with large differences between intraoperative exploration results. Hartmann et al. (24) discovered that examining PLC injury with x-ray and CT bony parameters had low sensitivity and specificity. Rihn et al. (25) considered the presence of a high signal in MRI lipid suppression images as the basis of PLC injury, but the specificity of this method is only 68.4%, making accurate judgment difficult. When none of the techniques can precisely assess the condition of a PLC injury, physicians must depend on their subjective clinical experience. Concurrently, PLC questionable damage was awarded 2 points, and damage was assigned 3 points, which immediately contributed to a substantial rise in the overall score T. PLC as part of the morphological structure of the spine, in theory, should be evaluated as a whole morphology, and separate score should not be offered. Consequently, assessing PLC damage and bone structural damage separately may result in an overemphasis on the function of PLC and repetitive scoring (26).

#### Addition of the “disc injury status” subcategory

Compared to the TLICS system, the modified TLICS system added the subcategory “intervertebral disc injury”. The reliability and repeatability kappa values were 0.815 and 0.879, respectively, which were both completely credible. According to studies (27), when direct violence is applied to the human body quickly, intervertebral disc injury is often unavoidable, and the upper intervertebral disc of the injured vertebra is more likely to be injured than the lower intervertebral disc. And the vertebral body itself accounts for only 38% of the unstable factors after thoracolumbar fractures, while the remainder is attributed to the intervertebral disc structure. When the disc injury is mild, the disc tissue does not herniate into the injured vertebral body, and conventional posterior surgery can successfully restore the normal height of the injured vertebrae, correcting the posterior convexity deformity, and restoring the intervertebral space height by bracing and resetting, allowing the damaged disc tissue to heal. When the intervertebral disc tissue is severely damaged, some of the disc tissue herniates into the injured vertebral body, the osteogenic ability is reduced, the bone healing ability is poor, and there is a possibility of instability and recompression. Coupled with the limited self-repair function of the disc tissue, the patient has a higher possibility of delayed retroconvex deformity (28–30). As a result, it is simple to conclude that spinal stability and intervertebral discs are related. According to the imaging characteristics of intervertebral disc injury, the modified TLICS system combined with MRI imaging data was therefore analyzed and classified into no injury, mild injury, and moderate-to-severe injury, and assigned 0, 1, and 2 scores, respectively. These scores, when combined with the other three subcategories, could aid in determining the severity of the fracture and providing clinical treatment.

## Conclusion

In conclusion, the modified TLICS system is intuitive and easy to use. When compared to the TLICS system, the modified TLICS system lowered the PLC integrity score and made treatment plan selection more objective. The addition of disc injury status subcategories, focusing more on the long-term stability of the spine, will assist clinicians in treating thoracolumbar fractures clinically and determining prognosis. Nonetheless, this is a retrospective, single-center study with a limited sample size. Moreover, given the limited size of our hospital, the lack of sample size calculation in this study weakens the trustworthiness of our findings. Therefore, with a larger sample size and perspective, multicenter research to confirm its clinical usefulness is required to conduct a more scientific evaluation and improve this method system.

## Data Availability

The original contributions presented in the study are included in the article/Supplementary Material, further inquiries can be directed to the corresponding author/s.
